# Cascade Desolvation and Conversion Reaction Driven by Self‐Tandem Catalysis for Wide‐Temperature Li‐S Batteries

**DOI:** 10.1002/advs.78441

**Published:** 2026-10-25

**Authors:** Jing Zhang, Shikai Yin, Mannan Yang, Hui Li, Caiyin You, Na Tian, Zhenjiang Cao, Yongzheng Zhang, Ruixian Duan, Hongzhen Lin, Jian Wang

**Affiliations:** ^1^ School of Materials Science and Engineering & Packaging Engineering and Digital Media Technology Xi'an University of Technology Xi'an China; ^2^ School of Chemistry Engineering Research Center of Energy Storage Materials and Devices Xi'an Jiaotong University Xi'an Shaanxi China; ^3^ School of Textile & Clothing Nantong University Nantong P. R. China; ^4^ i‐Lab Suzhou Institute of Nano‐tech and Nano‐bionics Chinese Academy of Sciences Suzhou China; ^5^ Helmholtz Institute Ulm (HIU) Ulm Germany; ^6^ Karlsruhe Institute of Technology (KIT) Karlsruhe Germany

**Keywords:** cascade reaction kinetics, interfacial desolvation, lithium sulfur battery, self‐tandem catalysis, shuttling inhibition

## Abstract

Dissatisfactory electrochemical performance of wide‐temperature lithium‐sulfur batteries (LSBs) originates from the sluggish Li^+^ desolvation and successive sulfur specie conversion kinetics at the interface and within the cathode interior. Herein, different from electrolyte engineering, a self‐tandem catalysis enabled cascade reactions is developed to drive Li^+^ kinetics and associated polysulfide conversions in wide‐temperature LSBs. By incorporating electron‐adjustable atom‐level Ce as the self‐tandem catalyst onto defect‐rich Co, Fe‐based layered double hydroxides (EAAC@D‐LDH), a favorable solvation microenvironment is established to decrease the related barriers toward promoting rapid Li^+^ desolvation, sulfur conversion, and Li_2_S delithiation kinetics, as revealed in Raman analysis, electrochemical evaluations, and ex‐situ interfacial morphology as well as composition characterization. Such improved reaction kinetics enable the sulfur cathodes work well in a wide temperature ranging from 0 to ‐30°C, and it retains high‐capacity retention of 94% at 5C. Under ‐20°C, the cells remain the capacity of 85% at 0.1C, respectively. Remarkably, a stable capacity is reserved even at an ultralow temperature of ‐30°C, demonstrating that the substantial promise of this self‐tandem catalysis supports cascade reaction refinement strategy toward high performance wide‐temperature LSBs.

## Graphical abstract

A self‐tandem catalysis accelerating cascade reaction strategy is pioneered through constructing electron‐adjustable atom‐level Ce as the self‐tandem catalyst to lower energy barriers for fast Li^+^ desolvation and sequential sulfur conversion kinetics. The catalyzed cells operate effectively over a wide‐temperature range from 0 to ‐30 °C, delivering a high‐capacity retention of 94% at 5C and retaining 85% of the capacity even under ‐20 °C, respectively.

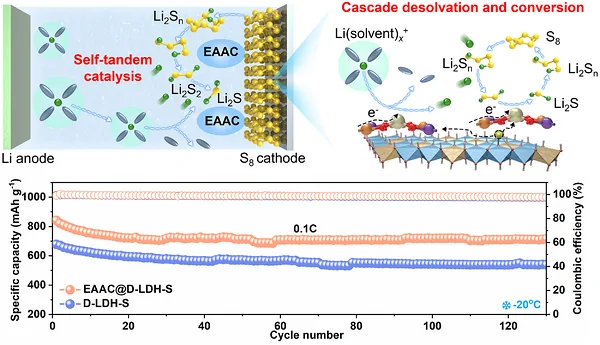

## 1 Introduction

The ever‐growing demand for high energy density reversible energy storage systems forces to develop new battery systems [1, 2, 3, 4, 5]. In comparison to lithium‐ion batteries, lithium‐sulfur batteries (LSBs) possess a higher theoretical energy density (2600 Wh kg^−1^) and benefit from the natural abundance of sulfur in the earth [6, 7, 8, 9, 10, 11]. However, LSB commercialization is severely hampered by soluble lithium polysulfides (Li_2_S*_x_*) shuttling, deteriorating the sulfur utilization and lifespan [12, 13, 14, 15]. In principle, sluggish interfacial Li(solvents)*_x_* ^+^ desolvation step impedes the consecutive sulfur redox processes of polysulfide conversions, and limited redox reversibility of Li_2_S delithiation leads to pronounced capacity fading and even failure [16, 17, 18, 19, 20]. Reducing the ambient temperature to subzero levels (below 0°C), the thermodynamic would be deactivate and the desolvation behavior of Li(solvents)*_x_* ^+^ becomes much more difficult since the solvation cluster is enlarged [21, 22, 23, 24, 25, 26], rendering the rate‐determining step of desolvation for successive sulfur redox reactions [27, 28, 29, 30, 31].

To tackle the above issues, common strategy is to construct conductive and porous hosts with integrated chemisorptive functionality to immobilize soluble polysulfides [1, 32, 33, 34, 35, 36, 37, 38]. Although it effectively suppresses shuttle effect, it is still plagued by sluggish ion transport in the sulfur conversions and Li_2_S delithiation. Fundamentally, in a kinetics view, Li^+^ transport kinetics involve three sequential steps from the bulk electrolyte, interfacial desolvation, and finally to the electrode interior, constituting a tandem reaction process with correspondingly energy barriers [12, 39]. The Li^+^ behaviors are governed by the chemical surroundings and it exhibits enhanced solvation phenomenon owing to the increasing volume of solvation shell under low temperature, which in turn raises the desolvation barrier at the interface.

Before the sulfur redox reaction can proceed, the solvated Li(solvents)*_x_* ^+^ cluster must be dissociated at the electrode/electrolyte interface to take off the solvent shells to release free Li^+^ [40, 41]. Despite pore engineering of employing metal organic frameworks (MOFs) or covalent organic frameworks (COFs) [42, 43, 44, 45, 46], incomplete dissociated Li(solvents)*_x_* ^+^ clusters at low temperatures fail to pass through the smaller porous structure solely via physical sieving. Obviously, decreasing the Helmholtz free energy barrier (ΔF) through electrocatalysis is beneficial for promoting the self‐diffusion kinetics of Li^+^ ion due to the enhanced hopping rate of ions from active sites to lattice, facilitating the release and accelerating the diffusion kinetics of free Li^+^ ions [47, 48].

As known, isolated atomic catalysts exhibit a high degree of electron delocalization that redistributes electron density for generating more active sites [7, 49, 50, 51, 52, 53], which is beneficial for Li^+^ desolvation due to enhanced electron accepting or donating abilities. Different from conventional three‐dimensional nanoparticles, two‐dimensional layered double hydroxides (LDH) have the advantages in tuning intrinsic *d*‐band electronic structure of Ni‐, Fe‐, or Co‐based LDH, which can facilitate interfacial charge transfer to establish a strong metal‐support electron interaction for stable atoms anchorment to provide more additional catalytic sites [54, 55]. Therefore, it is imperative to develop highly active and durable electrocatalysts of LDH‐stabilized metal atoms to reduce the cascade desolvation and reaction barriers under low temperature. At the same time, the relationship between self‐tandem catalytic effect and the cascade desolvation/diffusion behaviors, sulfur conversion reactions and Li_2_S delithiation needs to be comprehensively understood.

Herein, a self‐tandem catalysis accelerating cascade reaction strategy is pioneered to reconstruct the solvation microenvironment to lower the related Li^+^ desolvation and sulfur conversion barriers in wide‐temperature LSBs through incorporating electron‐adjustable atom‐level Ce as the self‐tandem catalyst onto defect‐rich Co, Fe‐based layered double hydroxides (EAAC@D‐LDH), as revealed in Figure 1A. As the representative demonstration, the combined detection of aberration‐corrected high‐angle annular dark‐field scanning transmission electron microscopy (HAADF‐STEM) and X‐ray photoelectron spectroscopy (XPS) has been used to visualize the presence of Ce atom and disclose the coordination bonds in the nanocomposites. Various characterizations of Raman analysis, electrochemical evaluation and ex‐situ interfacial morphology and composition analyses have comprehensively demonstrated that the as‐designed self‐tandem catalyst EAAC@D‐LDH on the cross‐linked electron network allows highelectrocatalysis efficiency to cooperatively accelerate the rate‐determining step of Li^+^ desolvation/diffusion, and sequential sulfur conversions, Li_2_S delithiation at both the electrode/electrolyte interface and within the cathode interior, ultimately suppressing shuttle effect and offering high‐capacity in wide‐temperatures LSBs. Consequently, the EAAC@D‐LDH catalyzed sulfur cathodes deliver a high capacity of 842 mA h g^−1^ at 0.1 C along with 85% capacity retention and a high‐rate performance up to 1C under ‐20°C, respectively. More impressively, the sulfur cathode still stabilizes at 0.1C even at an ultra‐low temperature of ‐30°C, confirming the fast kinetics of interfacial Li^+^ desolvation/diffusion and sulfur conversion with negligible polysulfide shuttling in low temperature LSBs.

**FIGURE 1:**
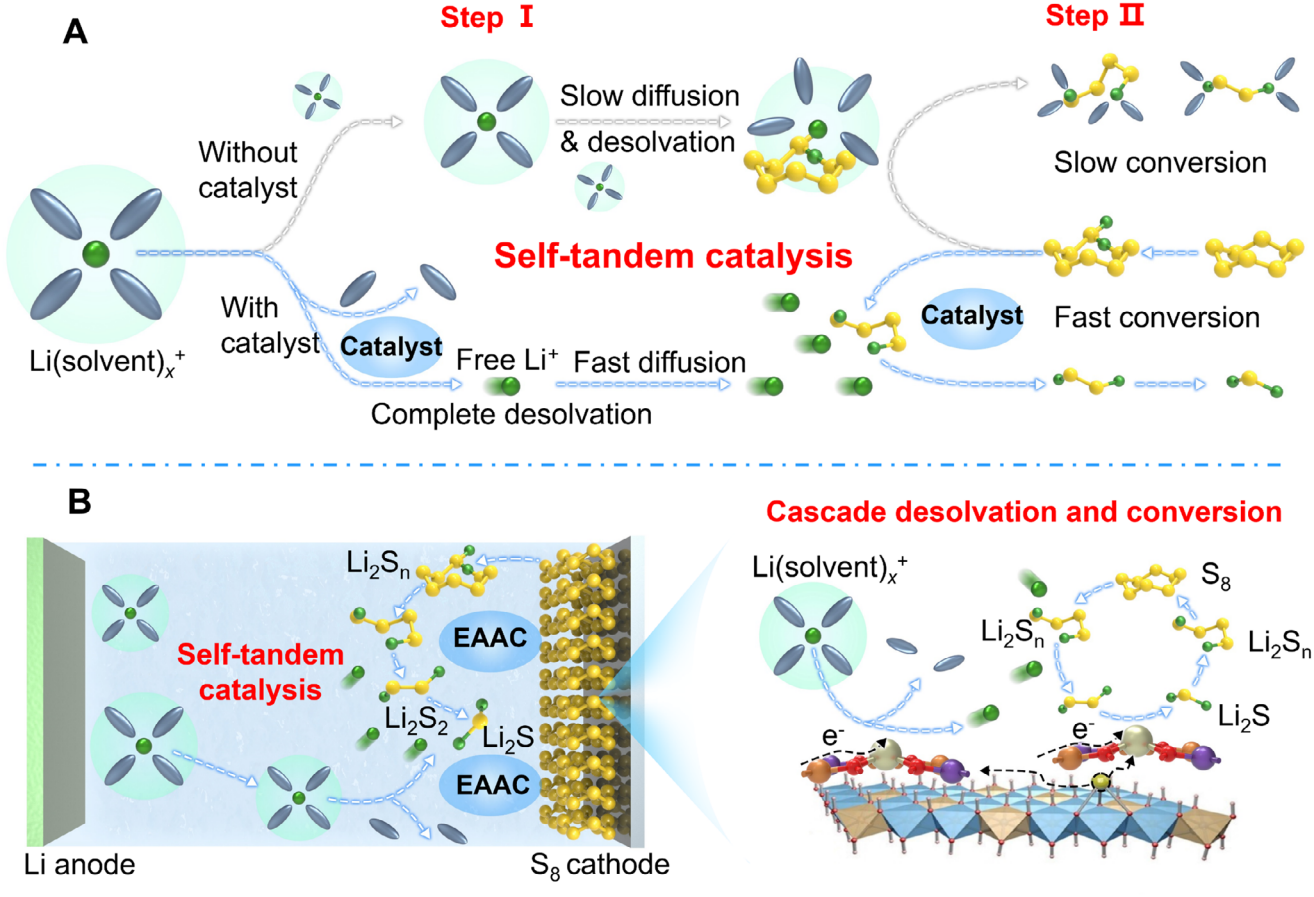
Schematic illustrations of Li^+^ and sulfur species kinetics at electrolyte/cathode interfaces. (A) Rapid cascade desolvation and sulfur conversion at the electrode/electrolyte interface with/without the self‐tandem catalyst; (B) Accelerated Li^+^ desolvation and sequential sulfur redox reactions are achieved by the EAAC@D‐LDH self‐tandem catalyst.

## 2 Results and Discussion

### 2.1 Structural and Morphological Characterization of the EAAC@D‐LDH as a Self‐Tandem Catalyst

The preparation of single‐atomic Ce anchoring on a cobalt (Co) iron (Fe)‐based layered defect‐rich double hydroxides (EAAC@D‐LDH) catalyst generally entails a two‐step process of initial hydrothermal synthesis and subsequent low‐temperature reduction, which benefits the delocalized electrons redistribution at the atom‐defect interface for successively accelerating the cascade reactions of Li^+^ desolvation, Li^+^ diffusion and sulfur redox reaction (Figure 1B). X‐ray diffraction (XRD) detection in Figure S1 shows clearly that the EAAC(3%)@D‐LDH possesses an identical crystalline structure and phase composition to the D‐LDH (with the atomic ratio of Co:Fe = 3:1, Ce: 3 wt %). Increasing the EAAC content from 1 wt.% to 5 wt.%, no Ce agglomeration species are detected. At the same time, no other impure peaks can be observed on the patterns, indicating no structure transformation arising from the defect formation and Ce atoms incorporation. As a demo, the nanocomposite containing 3 wt.% EAAC is denoted as EAAC@D‐LDH throughout this work. As observed in the SEM images (Figures S2 and S3), the as‐synthesized EAAC(1∼5%)@D‐LDH exhibits a rough and porous surface morphology without agglomeration of D‐LDH nanosheets. The surface areas of D‐LDH and EAAC@D‐LDH are 129.65 and 125.59 m^2^ g^−1^, respectively, with pore size centered around 40–70 nm (Figure S4), which further confirms that the introduction of EAAC exerts negligible impact on the porous morphology of the D‐LDH. Such phenomenon was further confirmed by the transmission scanning electron microscopy (TEM) images for the EAAC@D‐LDH, as shown in Figure 2A–E. The TEM images reveal the presence of nanosheets distributed on the carbon nanotubes surface (Figure 2A and Figure S5), identified as the D‐LDH with an interplanar spacing of 0.237 nm, corresponding to the (015) crystal plane (Figure 2B,C). In the selected area electron diffraction (SAED) pattern (Figure S6), diffraction patterns of (104) and (113) rings are evident, indicating the polycrystalline nature. As disclosed by energy dispersive X‐ray (EDX) detections, the homogeneous spatial distribution of elemental Ce, Fe Co and O throughout the EAAC@D‐LDH (Figure 2D and Figure S7) is observed, indicting the successful anchoring of elemental Ce. Furthermore, the atomic‐level dispersion of Ce species on the D‐LDH substrate was corroborated by aberration‐corrected high‐angle annular dark‐field scanning transmission electron microscopy (HAADF‐STEM). As shown in Figure 2E, numerous isolated bright dots are clearly resolved to individual Ce atoms without obvious clusters or nanoparticles, confirming that the Ce single atoms are atomically dispersed and stably anchored on the substrate.

**FIGURE 2:**
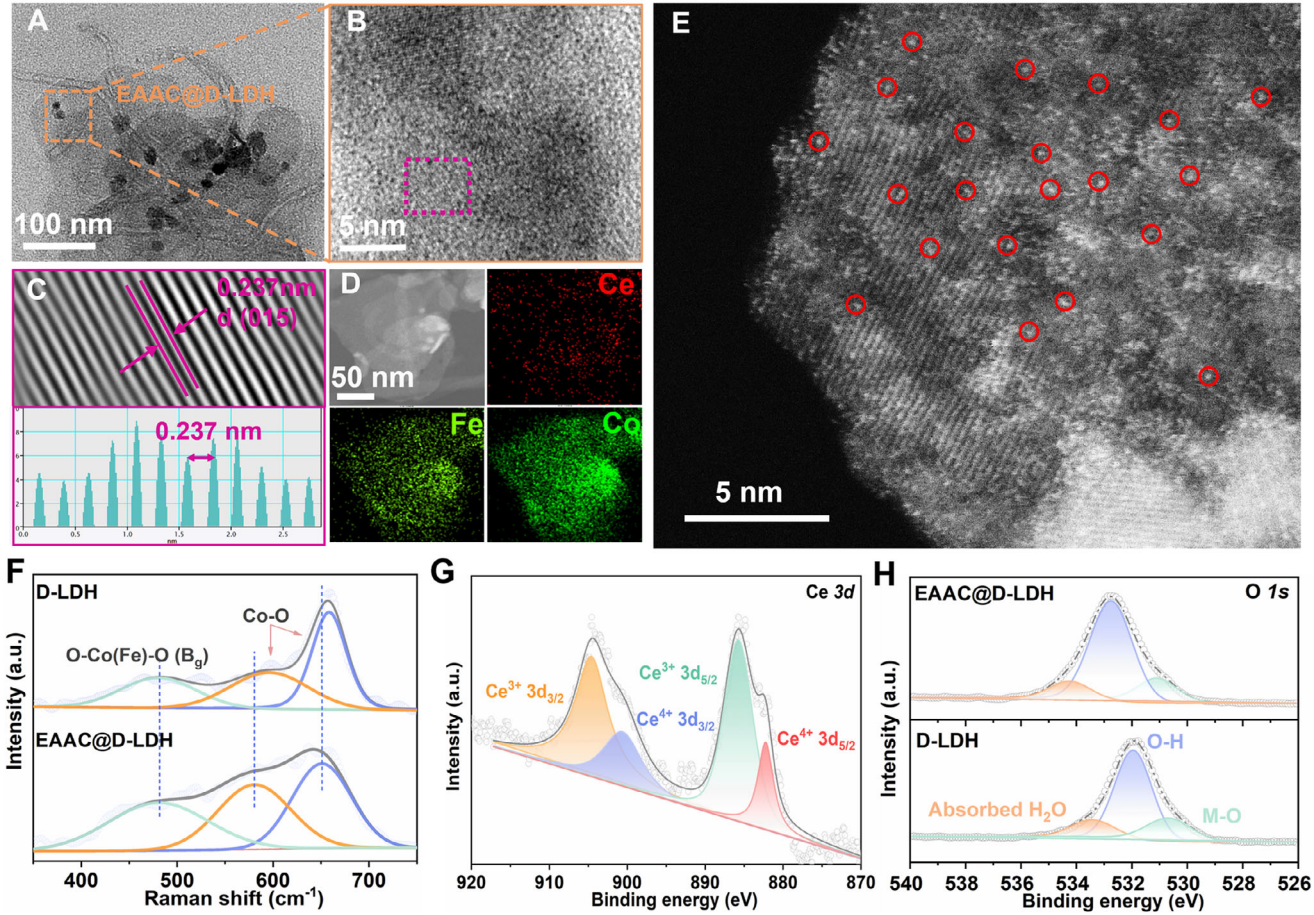
Atomic morphology and structural characterizations on the EAAC@D‐LDH nano catalyst. (A) TEM image and (B) HRTEM image of the EAAC@D‐LDH nanocomposite; (C) Fingerprint of interplanar spacings originated from IFFT images of the highlighted area of the EAAC@D‐LDH nanocomposite; (D) EDX elemental maps for the EAAC@D‐LDH including elemental Ce, Fe and Co. (E) HAADF‐STEM image of the EAAC@D‐LDH nanocomposite; (F) Raman spectra of O‐Co (Fe)‐O (B_g_) and Co‐O band in the resultant EAAC@D‐LDH and D‐LDH nanocomposites; High‐resolution XPS spectra comparison of (G) Ce 3d and (H) O 1s between the EAAC@D‐LDH and D‐LDH nanocomposites.

The Raman spectra of the I_D_/I_G_ ratio for the EAAC@D‐LDH present a minor increase from 1.24 to 1.28 (Figure S8). Also, the vibrational modes of at around 478 cm^−1^ are assign to O‐Co (Fe)‐O (B_g_) in the CoFe‐LDH species [55]. After incorporation Ce atoms, a distinct blue shift of this peak along with the Co‐O peaks located at 579 and 651 cm^−1^ is observed, suggesting that interactions between the incorporated Ce atoms and crystal defects leads to an electronic density redistribution (Figure 2F). As displayed in Figure S9 and 2G, the high‐resolution XPS spectrum of Ce 3d deconvoluted into four peaks of 904.64, 900.76, 885.73, and 882.25.2 eV is assigned to the pairs of Ce^3+^ 3d_3/2_/Ce^4+^ 3d_3/2_ and Ce^3+^ 3d_5/2_/Ce^4+^ 3d_5/2_ states, respectively, indicating atomic signatures of adjustable electron valence [56, 57]. In the high‐resolution O1s spectra (Figure 2H and Table S1), the decrease of the ratio of M‐O bonds (530.66 eV) along with the apparent blue shift peaks implies the successful anchoring of Ce atoms in the D‐LDH through M‐O‐Ce coordination bonds. Meanwhile, the observed positive shifts in the Co^2+^/Co^3+^ and Fe^3+^ XPS peaks (Figure S9) upon atomic Ce implanting suggest a strong Ce‐Co/Fe interaction in the EAAC@D‐LDH nanocomposite through a M‐O‐Ce (M = Co/Fe) bond, as demonstrated in the O1s XPS spectrum [54, 58].

### 2.2 EAAC@D‐LDH Catalyzed Interfacial Desolvation for Boosting Sulfur Redox Kinetics

The self‐tandem reactions comprise interfacial desolvation and sulfur conversion, where it requires fast dissociation of solvated Li^+^ into free Li^+^ before participating in redox reactions [28, 39]. Such desolvation step seems to be a coordination reaction with high barrier due to a reconstruction of the Li^+^ solvation sheath at the interface, which involves three sequential Li^+^ transport steps from the bulk electrolyte, interfacial desolvation, diffusion across the electrolyte/electrode interface and finally to the electrode interior. Upon the self‐tandem EAAC@D‐LDH catalyst, the inner Helmholtz plane (IHP) layer within the electrical double layer (EDL) will be altered by electron distribution (Figure 3A). According to the Schottky effect [59, 60], the EAAC@D‐LDH enables the IHP to enhance both uniformity and intensity of the micro‐electric field, benefiting the transfer of originally restricted “bound state” ions into high‐throughput “migration state” ions. However, the solvated Li ions fill the outer Helmholtz plane (OHP) layer and dissociate after entering the inner Helmholtz plane (IHP) layer without catalyst (Figure S10), slowing down the related redox reactions [61]. The ex‐situ Raman spectra in Figure 3B schematically display the state changes of Li(solvents)*_x_* ^+^ species at the electrode/electrolyte interface with/without EAAC@D‐LDH catalyst. Due to the lowered barrier of solvent rearrangement by the EAAC@D‐LDH in the desolvation step, a close association is indicated by the distinct spectral signatures of contact ion pairs (CIP) and aggregates (AGG) observed at 748–757 cm^−1^, which points to the existence of solvated solvent molecules at the interface (Figure 3B,C) [4, 17, 62, 63]. The elevated CIP/AGG intensity ratio suggests that desolvation proceeds to a greater extent, resulting in a high concentration of free Li^+^. The total intensities of AGG and CIP are higher in the EAAC@D‐LDH system (81%) than that on the D‐LDH (68%) and CNTs (53%) layer, indicating a higher concentration of free Li^+^ resulted from the enhanced desolvation capability catalyzed by EAAC@D‐LDH, as further illustrated in Figure 3D. Thereby, this reinforced desolvation allows an improvement of ion conductivity corresponding to approximately 34% increase of Li^+^ transport capability, rising from 1.66 mS cm^−1^ for the D‐LDH to 2.23 mS cm^−1^ for the EAAC@D‐LDH (Figure 3E and Figure S11). The rapid desolvation capability of EAAC@D‐LDH endows it with high chemical lithiophilicity, leading to effective chemisorption of soluble polysulfides through the disruption of the solvent sheath, as evidenced by the nearly colorless solution and the minimal ultraviolet visible (UV‐vis) absorbance at approximately 420 nm in Figure 3F. In the XPS analysis, the obvious positive shifts of S 2p peaks to higher binding energy for the EAAC@D‐LDH nanocomposite are obviously observed after the Li_2_S_8_ soaking in comparison to that of the D‐LDH (Figure 3G,H) [64]. The terminal sulfur/bridging sulfur (S_B_/S_T_) ratio showed a significant decrease from 6.4 (D‐LDH) to 3.6 (EAAC@D‐LDH), which confirms a more intensive chemical interaction between EAAC@D‐LDH and polysulfides (Figure 3H). Meanwhile, a newly emerged Ce^4+^3d_3/2_ peak (916.22 eV) in Figure 3I can be explained by the fact that the bridging S atoms from polysulfides are chemically interacted on the EAAC@D‐LDH catalyst to form Ce‐S bonds.

**FIGURE 3:**
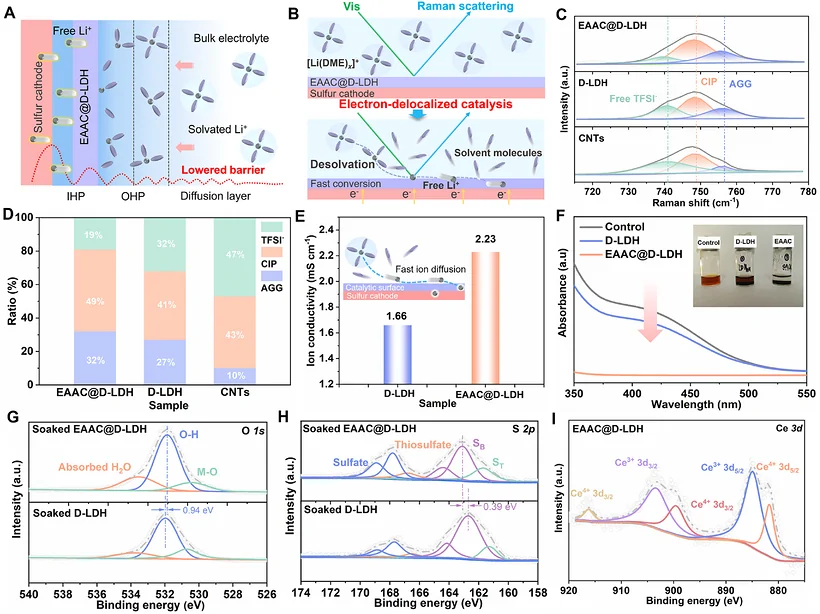
Characterization of Li^+^ desolvation and polysulfides behaviors catalyzed by EAAC@D‐LDH. (A) Interfacial evolution of the inner Helmholtz plane near the sulfur cathode regulated by EAAC@D‐LDH catalyst; (B) Schematic description of molecular states of Li^+^ solvation structure at the electrode/electrolyte interface with and without EAAC@D‐LDH catalyst under Raman analysis and (C) their corresponding Raman spectroscopy information; (D) The ratio summary on the free TFSI^−^, CIP, and AGG in different interfacial systems; (E) Li^+^ conductivity in different interfacial states; (F) UV‐vis spectra of the polysulfides solution after exposure to the absorbents for 6 h; High‐resolution XPS spectra of (G) O 1s, (H) S 2p and (I) Ce 3d in the EAAC@D‐LDH and D‐LDH nanocomposites after soak ing in 5 mmol L^−1^ Li_2_S_8_ solution.

### 2.3 Electrochemical Performance of Li‐S Battery Enabled by EAAC@D‐LDH Catalyst

The electrocatalytic activity of EAAC@D‐LDH in Li‐S batteries was systematically evaluated. Initially, electrochemical impedance spectroscopy (EIS) (Figure S12) of the EAAC@D‐LDH‐based cell reveals a reduced charge transfer resistance (13.4 vs. 30.7 Ω) along with a higher ion diffusion kinetics (7.01 vs. 10.64). Then, due to the self‐tandem catalysis of the EAAC on promoting Li^+^ desolvation and subsequent sulfur conversions kinetics, the cyclic voltammetry (CV) curves exhibit a positive shift alongside a smaller voltage polarization (ΔE) and stronger current peaks for the EAAC@D‐LDH‐S cathode (Figure 4A). The following excellent repeatability observed in the CV curves confirms the high reversibility of the EAAC@D‐LDH‐S cathode (Figure S13). Meanwhile, the EAAC@D‐LDH‐S cathode displays the smallest Tafel plot slope corresponding to the largest exchange current density (Figure S14). And the activation energy (Ea) for EAAC@D‐LDH‐S cathode decreases markedly by 39 and 110 kJ mol^−1^ in comparison to that with the D‐LDH‐S cathode, respectively (Figure 4B) due to the cascade catalysis effect [65, 66]. Thanks to the rapid interfacial Li^+^ desolvation, the CV curves and the corresponding contour plots of the EAAC@D‐LDH‐S depending on various scan rates show significantly enhanced current response compared to the control sample (Figure 4C,D and Figures S15 and S16), particularly within the voltage regions related with the conversion of Li_2_S_2_ or Li_2_S to Li_2_S*x* and final to S_8_. As a result, the EAAC@D‐LDH‐S cathode exhibits a considerably higher slope during the reduction process, reflecting an improved Li^+^ migration/diffusion rate (Figure 4E). Notably, the galvanostatic intermittent titration technique (GITT) on the EAAC@D‐LDH‐S cathode displays a substantially reduced voltage hysteresis between the nucleation and the activation compared with the D‐LDH‐S counterpart, confirming that the introduction of self‐tandem catalytic EAAC significantly facilitates lowering the initial desolvation energy barrier to assist the subsequently Li^+^ transportation for accelerated Li_2_S nucleation/activation processes (Figure 4F and Figure S17). Meanwhile, the EAAC@D‐LDH‐S cathode exhibits a lower internal reaction resistance (ΔR_internal_, Δ *R_internal_* = |Δ*V*_*QOCV* − *CCV*_| /*I_applied_*) at both the Li_2_S nucleation and activation stages, which reveals that the enhanced charge and mass transfer kinetics for facilitating efficient sulfur redox reactions (Figure 4G,H) [12].In‐situ EIS tests show better recovery to its initial resistance after completion of charging further reflects the better reversibility of the entire charge‐discharge cycle of the cathode with EAAC@D‐LDH (Figure 4I and Figure S18).

**FIGURE 4:**
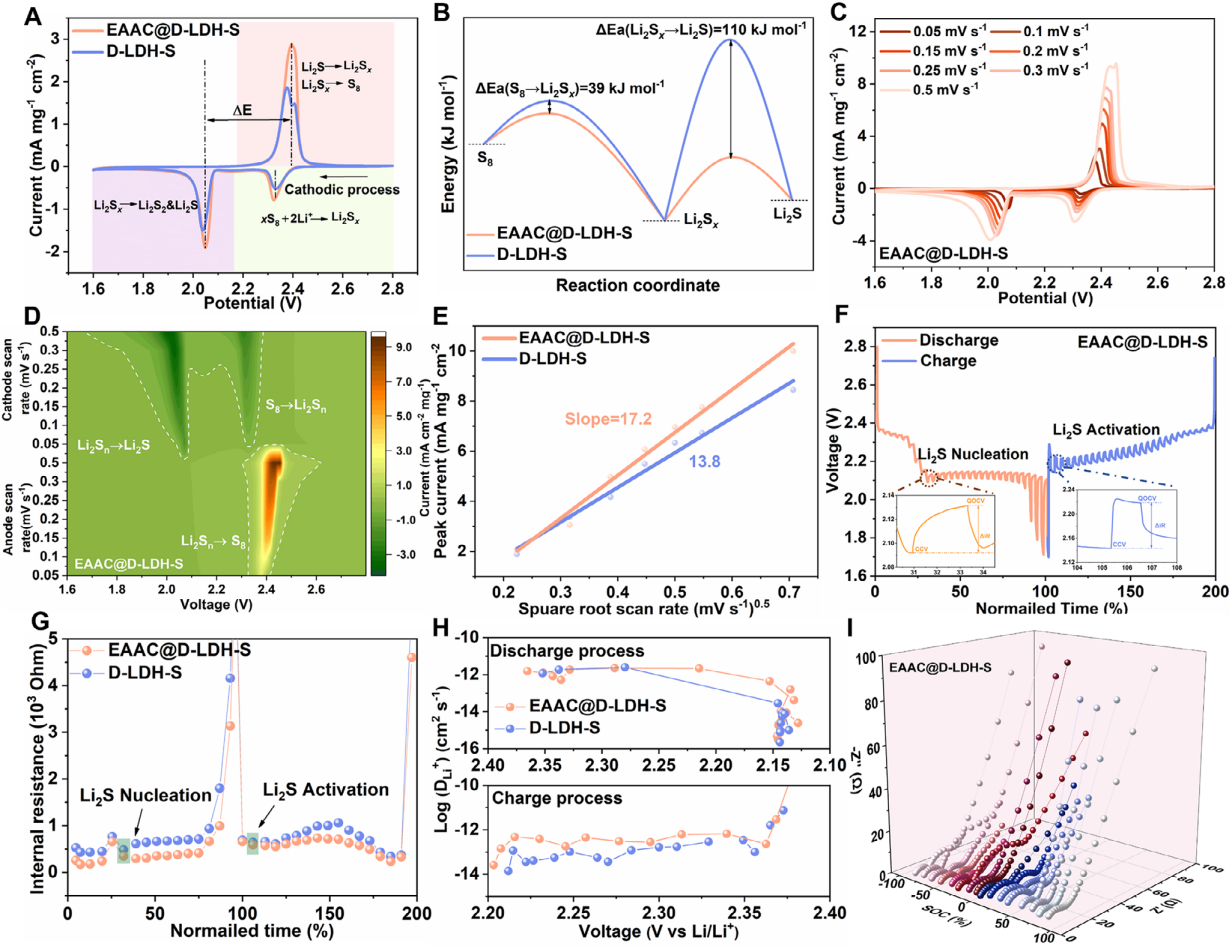
Fast desolvation enhanced the associated sulfur conversion kinetics of the EAAC@D‐LDH‐S cathode. (A) CV curves of Li‐S batteries with EAAC@D‐LDH and D‐LDH at a scan rate of 0.1 mV s^−1^; (B) The reaction activation energy differences derived from CV curve of the two sulfur cathodes; (C) Scan rate‐dependent CV curves and (D) the corresponding contour plots collected on the EAAC@D‐LDH‐S cathode (0.05‐0.5 mV s^−1^); (E) The linear relationship between I_p_ and (mV s^−1^)^1/2^ of LSBs with EAAC@D‐LDH and D‐LDH; (F) GITT plots and (G) the calculated ΔR_internal_ normalized to a discharge and charge cycle recorded on the EAAC@D‐LDH‐S cathode; (H) Log (D_Li_^+^) calculated from the GITT results; (I) In‐situ EIS measurement on the cell with EAAC@D‐LDH‐S cathode.

To further evaluate the of self‐tandem catalysis effect, the electrochemical performance of the temperature‐dependent EAAC@D‐LDH‐based cells was measured across a wide‐ temperature range. The concentration of EAAC contents ranging from 1 to 5 wt.% is investigated, where the EAAC(3%)@D‐LDH‐S cathode (denoted as EAAC@D‐LDH‐S throughout the work) exhibits the highest capacity and best stability (Figure S19). Decreasing to 0°C, the EAAC@D‐LDH‐S based cell exhibits a high reversible capacity of 493 mA h g^−1^ with a low decay rate of 0.056% per cycle at 0.5C (1C = 1675 mA g^−1^) and a Coulombic efficiency (CE) of nearly 100% after 400 cycles, in comparison to 0.08% for the D‐LDH‐S cathode cell (Figure 5A). Even decreasing the temperature to ‐10°C, the EAAC@D‐LDH‐S assisted cell still retains 90% of its initial capacity after 24 h of rest, significantly overwhelming the 79% retention observed for the D‐LDH‐S cathode (Figure 5B). Further lowering the temperature to as low as ‐20°C, the EAAC@D‐LDH catalyzed cell still delivers a high initial capacity of 842 mA h g^−1^ with a high‐capacity retention rate of over 85% after 130 cycles (Figure 5C,D), underscoring the excellent cycling stability. Shifting current rate from 0.1 to 1C, the EAAC@D‐LDH‐S cathode exhibits stable rate performance with well‐defined discharge plateaus compared to the D‐LDH‐S cathode (Figure 5E and Figure S20). Even at 1C, the EAAC@D‐LDH‐S cathode delivers higher capacity than the D‐LDH‐S (Figure 5E). Switching the temperature from 0 to ‐30°C (Figure 5F and Figure S21), the capacity of the EAAC@D‐LDH‐S electrode decreases gradually without any short‐circuit at each temperature whilst it maintains stable capacity of 303 mA h g^−1^ at 0.1 C even under ‐30°C, which is twice as large as the 151 mA h g^−1^ of the D‐LDH‐S electrode. In stark contrast, the D‐LDH‐S almost failed from ‐20°C to ‐30°C (Figure 5F,G and Figure S22) owing to the sluggish interfacial Li^+^ desolvation/diffusion kinetics. Overall, the EAAC@D‐LDH‐S based cells exhibited outstanding low‐temperature performance in comparison to the recently reported LSBs (Figure 5H and Table S2). Returning to room temperature, the EAAC@D‐LDH‐S cathode also displays a high‐rate performance up to 6 C (573 mA h g^−1^) under a consistently low overpotential (Figures S22 and S23) and retains a stable capacity of 799 mA h g^−1^ with a low‐capacity decay rate of 0.025% per cycle over 650 cycles at 3C (Figure S24). Even at 5C, a high initial capacity of 858 mA h g^−1^ is achieved, along with an exceptionally low‐capacity decay rate of 0.011% per cycle after 600 cycles (Figure 5I). In short, the EAAC@D‐LDH catalyst can lower the desolvation activation barriers of Li(solvent)*_x_* ^+^, thereby promoting solvent dissociation from solvation shell and releasing more free Li^+^, which in return facilitates the cascade Li^+^ diffusion and sulfur species conversions for high‐performance and wide‐temperature LSBs in a more energy‐favorable way.

**FIGURE 5:**
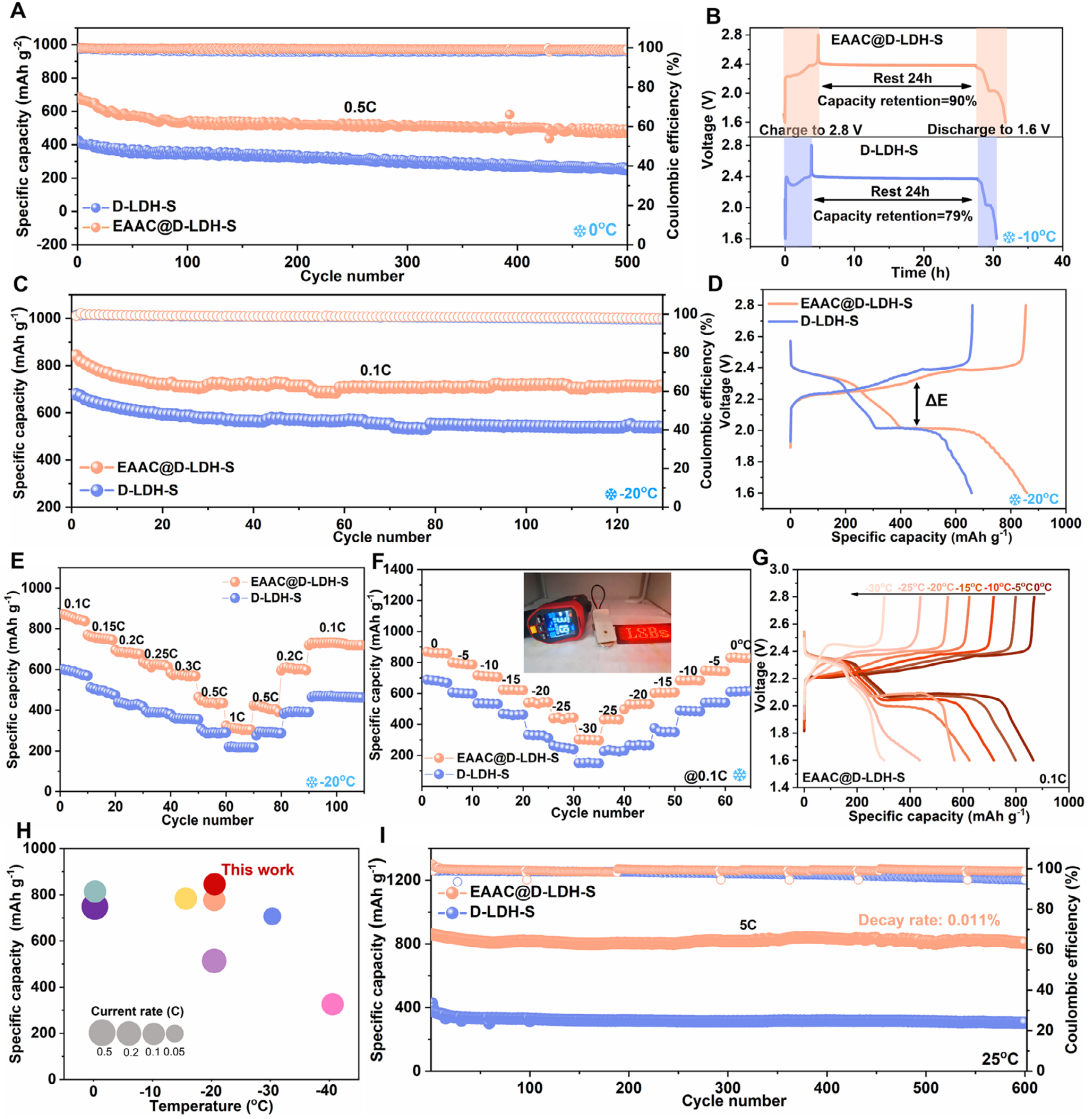
Low‐temperature electrochemical performances of Li‐S cells with EAAC@D‐LDH catalyst. (A) Cycle performance of the sulfur cathodes at 0.5 C under 0°C; (B) Self‐discharge behavior of Li‐S cells under resting for 24 h; (C) Cycle performance and (D) the corresponding initial charge‐discharge profiles of the sulfur cathodes at 0.1 C under ‐20°C; (E) The rate performance of two sulfur cathodes under ‐20°C; (F) Cycle performance of the two sulfur cathodes at 0.1 C; (G) Charge‐discharge profiles of the EAAC@D‐LDH catalyzed cell under variable‐temperature; (H) Performance comparison with previous reports; (I) Cycle performance of the sulfur cathodes at 5 C under room temperature.

### 2.4 Catalytic Mechanism Analysis of EAAC@D‐LDH

To gain deeper insight into the catalytic mechanism of the EAAC@D‐LDH, ex‐situ SEM, confocal laser scanning microscopy (CLSM), in‐depth XPS were performed. The top‐view and cross‐section SEM images together with the 3D CLSM image of the electrode after 100 cycles at 0.1C reveal that the EAAC@D‐LDH‐S cathode maintains a dense morphology with negligible volume expansion due to uniform Li^+^ flux from the accelerated desolvation process (Figure 6A,C,E), while the D‐LDH‐S cathode presents a looser and cracked structure resulted from repeated volume expansion due to the uneven Li^+^ flux during cycling (Figure 6B,D,F,G). Subsequently, the compositional changes of the sulfur cathode and cathode‐electrolyte interphase (CEI) were further assessed by in‐depth XPS, as presented in Figure 6H–N and S25‐S32. In detail, the sulfur content including S_8_ (∼163.4 and ∼164.8 eV) and LiPSs (∼161.8 eV) within the S 2p spectra for the EAAC@D‐LDH‐S cathode exhibits a progressive increase from approximately 6% to 50% with extended sputtering time (Figure 6I,K) [67]. Such upward trend suggests the strong catalytic ability of the EAAC@D‐LDH in promoting Li^+^ desolvation for fast sulfur conversions against shuttle effect and facilitating the encapsulation of most sulfur species. However, the D‐LDH‐S cathode exhibit a much stronger shuttle effect and thicker CEI due to the superficial sulfur of as high as 21%∼32% (Figure S26). Concurrently, a pronounced decrease of the F‐Li peak (∼57.65 eV) content in Li 1s spectra was observed (Figure 6J and Figure S27), along with a gradually attenuated Li‐F peak (∼684.94 eV) intensity in F 1s spectra (Figure S28), corroborating the formation of a LiF‐enriched CEI on the surface of the EAAC@D‐LDH‐catalyzed sulfur cathode [27] In contrast, the D‐LDH‐S cathode exhibits irregularly distributed F‐Li species in the Li *1s* and F *1s* spectra, indicative of a nonuniform and fractured CEI layer due to uncontrolled volumetric explosion during cycling (Figures S28 and S29), which is in good agreement with the ex‐SEM and CLSM images in Figure 6A–G. To better clarify the effect of self‐tandem EAAC@D‐LDH catalyst on inhibiting polysulfide shuttling, the peak deconvolution of S 2p spectra profiling from cycled separators reveals distinct contributions from Li_2_S_4_ (∼162.74 and ∼161.45 eV) and Li_2_S (∼160.12 eV) (Figure 6L and Figure S30) [68]. After sputtering, the Li_2_S signal associated with self‐discharge product drops abruptly to 0 (Figure 6L,N and Figure S31) [67]. Simultaneously, the Li 1s spectra in Figure 6M exhibit a marked attenuation of Li‐S species (∼52.42 eV). However, the D‐LDH‐S cell provides an initially 1.7‐fold higher Li_2_S content (23.7% vs. 13.7%) (Figures S31 and S32), collectively suggesting a severe shuttling effect of LiPSs.

**FIGURE 6:**
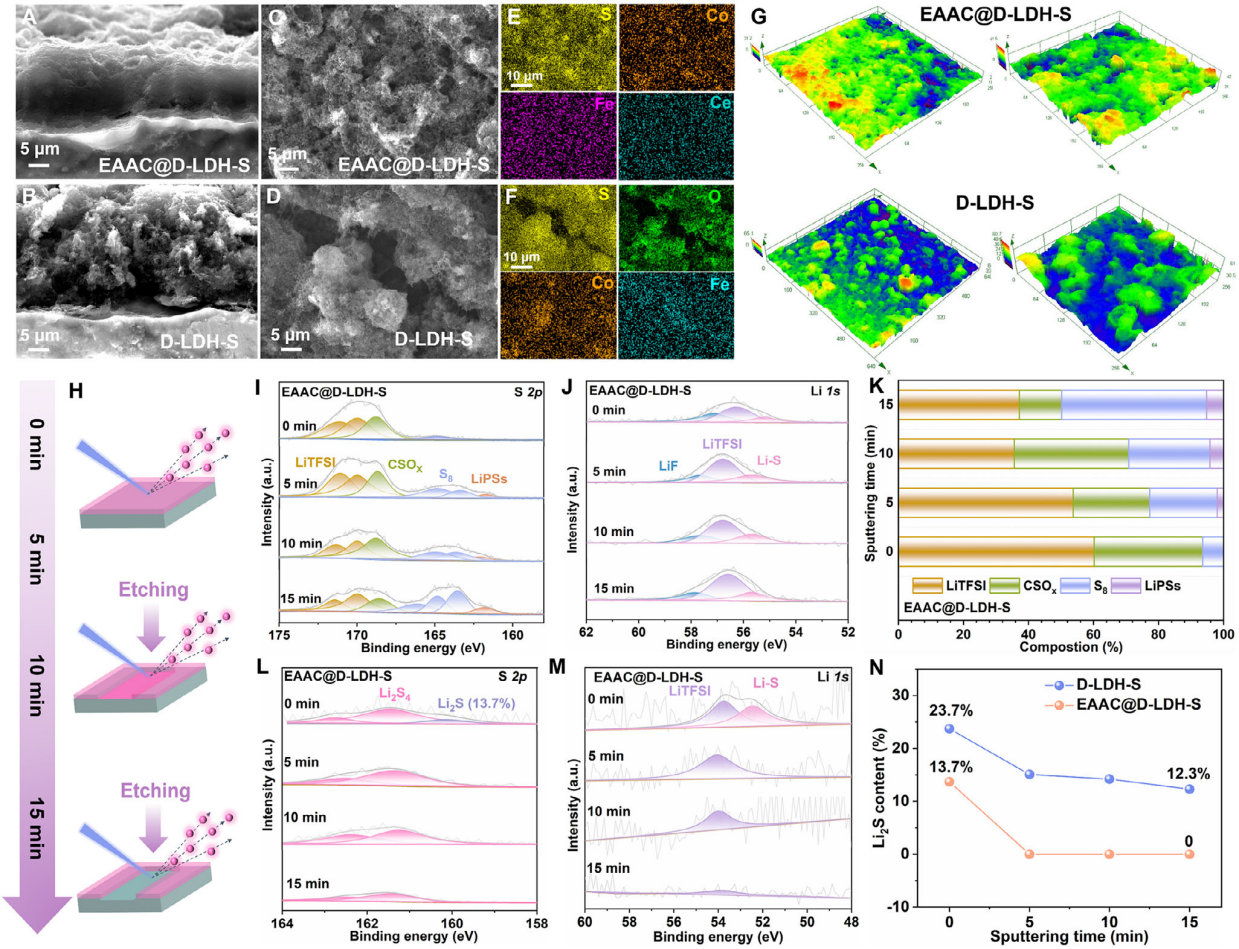
Mechanism analysis of the EAAC@D‐LDH catalyst in regulating Li^+^ desolvation and related sulfur conversion behaviors. (A‐F) SEM and EDS elemental mappings of the EAAC@D‐LDH‐S and D‐LDH‐S cathode after 100 cycles at 0.1C under ‐20°C; (G) Confocal microscopic 3D morphology of the EAAC@D‐LDH‐S and D‐LDH‐S cathode after the cycling; (H) Schematic illustration of the in XPS depth profiling of CEI formed on the cathode/electrolyte interfaces or separators after cycling for 100 cycles at ‐20°C, including high‐resolution (H) S 2p and (J) Li 1s spectra on the sulfur cathodes and (K) the corresponding comparison of the component ratio of interfacial sulfur species on the cathode, high resolution (L) S 2p and (M) Li 1s spectra on the separators and (N) the corresponding comparison of Li_2_S content within the separators.

## 3 Conclusion

In summary, we have proposed a new self‐tandem catalysis stimulating cascade reaction strategy to address the sluggish Li^+^ kinetics and related shuttling effect in wide‐temperature LSBs. By rationally employing the self‐tandem catalyst of isolated atomic Ce anchored on the defect‐rich Co, Fe layered double hydroxides (EAAC@D‐LDH), the fast Li^+^ desolvation/diffusion, sulfur conversions and Li_2_S delithiation kinetics at the electrolyte/cathode interface and within the cathode interior are achieved. The pivotal catalytic roles and underlying mechanism of the EAAC@D‐LDH in modulating the solvation structure, accelerating Li^+^ transport and catalyzing polysulfides conversions by lowering the barriers are comprehensively characterized by desolvation Raman analysis, electrochemical evaluations and ex‐situ interfacial morphology and composition analyses. Consequently, the EAAC@D‐LDH refined desolvation and reaction kinetics significantly enable the sulfur cathodes to achieve a high‐capacity retention of 85% and robust capacity at ‐20 or ‐30°C, highlighting the substantial potential of the self‐tandem catalysis for next‐generation wide‐temperature LSBs.

## Author Contributions

**Jing Zhang**: investigation, Writing – original draft, Writing – review and editing, formal analysis, data curation, conceptualization, methodology. **Shikai Yin**: investigation, writing – original draft, visualization, software, methodology, formal analysis. **Mannan Yang**: methodology, investigation, formal analysis. **Hui Li**: investigation, formal analysis. **Caiyin You**: resources, writing – review and editing, methodology, supervision. **Na Tian**: methodology, resources. **Zhenjiang Cao**: methodology, software. **Yongzheng Zhang**: investigation, resources. **Ruixian Duan**: methodology. **Hongzhen Lin**: writing – review and editing, resources, funding acquisition. **Jian Wang**: conceptualization, methodology, writing – review and editing, supervision, resources, project administration, funding acquisition.

## Conflicts of Interest

The authors declare no conflicts of interest.

## Supporting information




**Supporting File**: advs78441‐sup‐0001‐SuppMat.docx.

## Data Availability

The data that support the findings of this study are available from the corresponding author upon reasonable request.
