# Supplementary material for: Cascade Desolvation and Conversion Reaction Driven by Self‐Tandem Catalysis for Wide‐Temperature Li‐S Batteries
Source: Adv Sci (Weinh). 2026 Oct 25:e78441. Online ahead of print. doi: 10.1002/advs.78441 (PMC13696881; doi:10.1002/advs.78441)
Supplement: Supplementary file 1 [file ADVS-9999-e78441-s001.docx]

**Cascade Desolvation and Conversion Reaction Driven by Self-Tandem Catalysis for Wide-Temperature Li-S Batteries**

*Jing Zhang^*^, Shikai Yin, Mannan Yang, Hui Li, Caiyin You^*^, Na Tian, Zhenjiang Cao, Yongzheng Zhang, Ruixian Duan, Hongzhen Lin^*^ and Jian Wang^*^*

J. Zhang, S. Yin, M. Yang, H. Li, C. You, N. Tian, R. Duan

School of Materials Science and Engineering & Packaging Engineering and Digital Media Technology, Xi’an University of Technology, Xi’an 710048, China

J. Wang

Helmholtz Institute Ulm (HIU), Ulm D89081, Germany; Karlsruhe Institute of Technology (KIT), Karlsruhe D76021, Germany

H. Lin

*i*-Lab, Suzhou Institute of Nano-tech and Nano-bionics, Chinese Academy of Sciences, Suzhou 215123, China

Z. Cao

School of Chemistry, Engineering Research Center of Energy Storage Materials and Devices, Xi’an Jiaotong University, Xi'an, Shaanxi 710049, China

Y. Zhang

School of Textile & Clothing, Nantong University, Nantong 226019, PR China

**Corresponding authors**: zhangjing2020@xaut.edu.cn

caiyinyou@xaut.edu.cn

hzlin2010@sinano.ac.cn

jian.wang@kit.edu; wangjian2014@sinano.ac.cn

**Experimental**

***Synthesis of*** ***EAAC@D-LDH nanocomposite***

The self-tandem catalyst of electron-adjustable atom-level Ce anchored onto defect-rich Co, Fe-based layered double hydroxides (EAAC@D-LDH) was synthesized through a combination of solvothermal method with successive low-temperature hydrogen treatment. Typically, hexahydrate cobalt nitrate (Co(NO_3_)_2_·6H_2_O, aladdin) of 873 mg and heptahydrate iron nitrate of 404 mg (Fe(NO_3_)_3_·9H_2_O, aladdin) were dissolved in deionized water (25 mL) with constant stirring, resulting in a clean yellow solution. Subsequently, 400 mg of purified multi-walled carbon nanotubes (CNTs, Cnano, China, Fltube 9000) were sonicated in 100 mL of deionized water for 1 h to obtain a uniform suspension. Thereafter, the clean yellow solution was added dropwise into the CNTs dispersion under mechanical stirring, followed by the addition of sodium hydroxide of 560 mg (NaOH, aladdin) and sodium carbonate of 636 mg (Na_2_CO_3_, aladdin). The formed mixture was transferred to a Teflon-lined stainless-steel autoclave and reacted at 95℃ for 36 h to yield the Co, Fe-based layered double hydroxides with CNTs hybrids (CF-LDH/CNTs) intermediate. The resultant CF-LDH/CNTs was then etched with 60 mL of formamide solution for 30 min, followed by filtration and washing by deionized water and anhydrous ethanol repeatedly to obtain the etched CF-LDH/CNTs. Subsequently, the etched CF-LDH/CNTs (200 mg) and 23.74 mg of cerium nitrate hexahydrate (Ce(NO_3_)_3_·6H_2_O, aladdin) was dispersed in 10 mL of anhydrous ethanol with continuous stirring for 12 h to from a homogeneous suspension. After rapidly removing the solvent by evaporation, the collected solid was reduced under a H_2_/Ar atmosphere (H_2_, 5% in volume) at 100°C for 2 h with a heating rate of 5℃ per minute, yielding the target product of EAAC@D-LDH. The defect-rich Co, Fe-based layered double hydroxides on CNTs nanocomposite (D-LDH) was synthesized using the same method without atomic precursors.

***Preparation of sulfur cathodes nanocomposites***

The as-synthesized EAAC@D-LDH (200 mg) and polyvinyl pyrrolidone (PVP, 20 mg) were ultrasonically dispersed in 600 mL of deionized water to form a uniform suspension. Separately, a Na_2_S_x_ solution was prepared by adding commercial sulfur powder (0.72 g) and Na_2_S·9H_2_O (1.82 g) to 25 mL of deionized water, followed by magnetically stirring until a solution was generated. The obtained Na_2_S_x_ solution was then slowly added into the aforementioned suspension under vigorous magnetic stirring. The HCOOH solution (2 mol L^-1^) was subsequently introduced, and the mixture was continuously stirred for 12 hours. The resultant product was then collected through filtration, rinsed with deionized water to eliminate soluble impurities. After freeze-dried for 24 h, the collected powder was ultimately sealed in a container under an argon atmosphere and heated at 155℃ for 12 h to afford the EAAC@D-LDH-S nanocomposite material with sulfur loading of 75 wt%.

***Polysulfide adsorption analysis***

0.092 g of Li_2_S (Aladdin) and 0.448 g sulfur (Aladdin) were dissolved into 10 mL of a DOL/DME mixed solvent (1:1 v/v, Suzhou, China) to form a homogeneous Li_2_S_8_ solution (0.2 mol L^-1^) through stirring. The 0.2 mol L^-1^ Li_2_S_8_ solution was then diluted to 0.005 mol L^-1^ with DOL/DME solvent. The diluted solution was dispensed into three screw-cap vials each containing 8 mL. EAAC@D-LDH and D-LDH absorbents (0.05 g each) were immersed into two of the vials for 8 h, separately. Subsequently, the color change of the solutions was recorded using a digital camera, and the absorbance was evaluated by ultraviolet visible (UV-vis) spectroscopy. All the operation performed were performed in a glove box filled with high pure argon.

***Cells assembly***

To prepare the sulfur composite cathode, the EAAC@D-LDH-S nanocomposite, carbon black and polyvinylidene fluoride (PVDF) binder were mixed in a weight ratio of 7:2:1 and dispersed in N-methylpyrrolidone (NMP) solvent. The mixture was stirred continuously to form a homogeneous slurry, which was then cast onto an aluminum foil current collector using a doctor blade with a controlled wet thickness of 150 μm. After drying in a vacuum oven at 60℃ for 24 h, the resultant electrode sheet was punched into circular discs with a diameter of 12 mm and a sulfur loading of ∼1.2 mg cm^-2^. A Li-S cell was assembled using the prepared discs as the cathode and lithium foil as the anode separated by a commercial Celgard 2350 separator, filling 1 mol L^-1^ LiTFSI and 1 wt% LiNO_3_ in 1,3-dioxolane/1,2-dimethoxyethane solution (DOL/DME, 1:1 in volume) as the electrolyte (15 μL mg^-1^). The electrolyte/sulfur (E/S) ratio is about 15 μL mg^-1^ per coin cell, and the cell tested under low temperature using the same electrolyte formulation as those at room temperature. The cell was assembled inside a glove box full of high pure argon gas (H_2_O ≤ 0.5 ppm, O_2_ ≤ 0.5 ppm, MBRAUN LABSTAR).

***Materials and devices characterization***

X-ray diffraction (XRD) patterns were collected on an XRD-7000S diffractometer with copper Kα radiation (λ= 0.15418 nm) over a 2θ range of 5^o^~70^o^. X-ray photoelectron spectroscopy (XPS) was carried out using an ESCALAB 250XI spectrometer to determine the elemental composition and coordination environment of the samples. Raman spectra were acquired on a Horiba laser Raman spectrometer (EUROVECTOR EA3000) to analyze the defective structure of composite materials. The morphology observation of as-prepared nanocomposite materials was examined using a scanning electron microscope (Zeiss MERLIN Compact). The atomic morphology and distribution were characterized by a spherical aberration-corrected transmission electron microscopy (JEM NEO ARM200CF). Electrochemical impedance spectroscopy (EIS) measurements were performed on an electrochemical workstation (VMP-3) to determine the electron/ion transfer state at the interface over a frequency range of 10^-2^ to 10^5^ Hz with an AC amplitude of 5 mV. Galvanostatic charge-discharge tests were carried out using the Neware Battery Tester (BTS-5 V 20 mA) to evaluate the Coulombic efficiency, cycling and rate performances of cells within the voltage range of 1.6-2.8 V vs. Li/Li^+^ electrode.

**Figure S1** XRD patterns of EAAC(1~5 wt%)@D-LDH with different content of atomic Ce and D-LDH nanocomposites.

**Figure S2** SEM images of the EAAC(1~5 wt%)@D-LDH with different content of atomic Ce and D-LDH nanocomposites.

**Figure S3** High-resolution SEM images of (A) the EAAC@D-LDH with 3wt% atomic Ce and (B) D-LDH nanocomposites.

**Figure S4** (A) Nitrogen absorption/desorption isotherms and (B) pore size distribution of the EAAC@D-LDH and D-LDH nanocomposites.

**Figure S5** TEM image of the D-LDH nanocomposite.

**Figure S6** Selected-area electron diffraction pattern of the EAAC@D-LDH nanocomposite.

**Figure S7** EDX elemental mapping of O for the EAAC@D-LDH nanocomposite.

**Figure S8** Raman spectra of D and G band in the resultant EAAC@D-LDH and D-LDH nanocomposites.

**Figure S9** (A) Comparison of the XPS survey spectra, high resolution (B) Co 2p and (C) Fe 2p spectra for the EAAC@D-LDH and D-LDH nanocomposites.

**Figure S10** Interfacial evolution of the inner Helmholtz plane (IHP) near the sulfur cathode induced by D-LDH.

**Figure S11** Ion conductivity measured on the EAAC@D-LDH and D-LDH.

**Figure S12** Comparison of EIS between the EAAC@D-LDH-S and D-LDH-S electrodes and (B) the corresponding linear relationship between Z′ and ω^-1/2^.

**Figure S13** CV curves of Li-S batteries with (A) EAAC@D-LDH and (B) D-LDH at a scan rate of 0.1 mV s^-1^.

**Figure S14** Tafel plots related to the reductions of elemental sulfur to (A) S_8_→Li_2_S*_x_* and (B) Li_2_S*_x_* →Li_2_S.

**Figure S15** Scan rate-dependent CV curves of the D-LDH-S cathode.

**Figure S16** Contour plot of CV curves with various scan rates (0.1-0.5 mV s^-1^) for the D-LDH-S cathode.

**Figure S17** GITT plots collected on the D-LDH-S cathode.

**Figure S18** In situ EIS measuring on the cell with D-LDH-S cathode.

**Figure S19** Cycle performance of the EAAC(1~5 wt%)@D-LDH-S and D-LDH-S cathodes at 1 C.

**Figure S20** Voltage-capacity profiles for (A) EAAC@D-LDH-S and D-LDH-S cathodes at -20^o^C.

**Figure S21** Voltage-capacity profiles of the D-LDH-S cathode at 0.1C under various temperature.

**Figure S22** The rate performance of the two sulfur cathodes under room temperature.

**Figure S23** Charge-discharge curves for (A) the EAAC@D-LDH-S and (B) D-LDH-S cathodes at room temperature.

**Figure S24** Cycle performance of the EAAC@D-LDH-S and D-LDH-S cathodes at 3C at room temperature.

**Figure S25** In-depth profiling XPS survey spectra of the cycled (A) EAAC@D-LDH-S and (B) D-LDH-S cathodes.

**Figure S26** (A) High-resolution S 2p spectra of XPS depth profiling on the cycled D-LDH-S cathode and (B) the corresponding comparison of each component.

**Figure S27** Comparison in the component of Li species based on the XPS depth profiling high-resolution Li 1s spectra of the cycled EAAC@D-LDH-S cathode.

**Figure S28** (A) XPS depth profiling high-resolution F 1s spectra and (B) the corresponding LiF content comparison obtained on the cycled EAAC@D-LDH-S and D-LDH-S cathodes.

**Figure S29** (A) XPS depth profiling high-resolution Li 1s spectra and (B) comparison in the component of corresponding Li species collected on the cycled D-LDH-S cathode.

**Figure S30** In-depth profiling XPS survey spectra of separators from the cycled cells based on (A) EAAC@D-LDH-S and (B) D-LDH-S cathodes.

**Figure S31** XPS depth profiling high-resolution (A) S 2p and (B) Li 1s spectra detected on the cycled cell based on D-LDH-S cathode.

**Figure S32** Comparison in the component of Li species based on the XPS depth profiling high-resolution Li 1s spectra detected from the separators of the cycled cells based on EAAC@D-LDH-S and D-LDH-S cathodes.

**Table S1** The ratio summary of absorbed H_2_O, M-OH and M-O bonds in different samples

| Samples | Absorbed H_2_O | M-OH bond | M (Fe, Co)-O bonds |
| --- | --- | --- | --- |
| EAAC@D-LDH | 13% | 71% | 16% |
| D-LDH | 14% | 65% | 21% |

**Table S2** Electrochemical performance of low-temperature Li-S cells from previous works.

| **Cathodes** | **Low-temperature performance (mAh g^-1^)** | **Room-temperature performance (mAh g^-1^)** | **References** |
| --- | --- | --- | --- |
| **EAAC@D-LDH-S** | **842 (-20 ℃, 0.1C, 130 cycles 85%)** | **873 (5C, 600 cycles 94%)**  **950 (3C, 650 cycles, 88%)** | **Our work** |
| CNF-S | 720 (-30 ℃, 0.05C,110 cycles 86%) | 646 (2C) | *Adv. Sci.* **2024**, 12, 2410628.[1] |
| KB/S with SAFe modified separator | 340 (-40 ℃, 0.1C, 100 cycles 92%) | 985 (1C, 600 cycles, 73%) | *Angew. Chem. Int. Ed.* **2022**, 61, e202211933. [2] |
| S/AB with GO-CoNiP modified separator | 810 (0 ℃, 0.1C, 120 cycles 84%) | 658(4C, 450 cycles 90%)  803(2C, 600 cycles, 60%) | *Small* **2023**, 20, 2307912.[3] |
| Li_2_S_6_@HDS-NCCNT | 502.3(-20 ℃, 0.2C, 85 cycles 82.8%) | 660(1C) | *Chem. Eng. J.* **2025**, 511, 161939.[4] |
| S/C with Fe/Ni-N@NC modified separator | 741(0 ℃, 0.5C, 300 cycles 74.8%) | 960(1C,1000 cycles 50%) | *Chem. Eng. J.* **2024**, 484, 149171.[5] |
| TCC-S | 790(-20 ℃, 0.1C, 80 cycles 75%) | 642(5C, 600 cycles 71%) | *J. Energy Chem.* **2025**, 105, 461.[6] |
| S/KB@CuS | 800(-15 ℃，0.1C ,100 cycles 88%) | 799 (1C)  1200 (0.2C,100 cycles 68%) | *Energy Storage Mater.* **2024**, 66, 103215.[7] |

**References**

[1]	Qi, Q., Shi, F., Yu, J., et al., "Understanding the Dynamics of Sulfur Droplets Formation in Lean‐Electrolyte and Low‐Temperature Lithium–Sulfur Batteries," *Adv. Sci.* 12, no. 4 (2024), <https://doi.org/10.1002/advs.202410628>.

[2]	Xu, J., Zhang, H., Yu, F., et al., "Realizing All‐Climate Li‐S Batteries by Using a Porous Sub‐Nano Aromatic Framework," *Angew. Chem. Int. Ed.* 61, no. 47 (2022), <https://doi.org/10.1002/anie.202211933>.

[3]	Li, J., Wu, X., Jian, C., et al., "GO‐CoNiP New Composite Material Modified Separator for Long Cycle Lithium–Sulfur Batteries," *Small* 20, no. 17 (2023), <https://doi.org/10.1002/smll.202307912>.

[4]	Shi, J., Yu, C., Ning, Y., Li, B., Wang, B., Zheng, S., "Strategic design of high-directional scaffold enabling accelerated kinetics for high-loading low-temperature lithium-sulfur batteries," *Chem. Eng. J.* 511(2025), <https://doi.org/10.1016/j.cej.2025.161939>.

[5]	Xu, Y., Zhu, Y., Nie, T., et al., "Interwoven n-doped bimetallic/carbon nanosheets as an efficient electrocatalyst for wide-temperature lithium-sulfur batteries," *Chem. Eng. J.* 484(2024), <https://doi.org/10.1016/j.cej.2024.149171>.

[6]	Xiao, Z., Fang, J., Ge, H., Gao, H., Wang, L., "Curvature-regulated sulfur electrochemistry for robust low-temperature Li-S batteries," *J. Energy Chem.* 105(2025), <https://doi.org/10.1016/j.jechem.2025.02.005>.

[7]	Jiang, R., Qi, X., Ji, J., et al., "Accelerated Li2S conversion in sparingly-solvating electrolytes enabled with dipole-dipole interaction for wide-temperature Li-S batteries," *Energy Storage Mater.* 66(2024), https://doi.org/10.1016/j.ensm.2024.103215.
